# New Anti-Inflammatory 9,11-Secosterols with a Rare Tricyclo[5,2,1,1]decane Ring from a Formosan Gorgonian *Pinnigorgia* sp.

**DOI:** 10.3390/md14120218

**Published:** 2016-11-26

**Authors:** Yu-Chia Chang, Tsong-Long Hwang, Jyh-Horng Sheu, Yang-Chang Wu, Ping-Jyun Sung

**Affiliations:** 1 National Museum of Marine Biology and Aquarium, Pingtung 944, Taiwan; jay0404@gmail.com; 2 Graduate Institute of Natural Products, College of Medicine, Chang Gung University, Taoyuan 333, Taiwan; htl@mail.cgu.edu.tw; 3 Research Center for Industry of Human Ecology, Research Center for Chinese Herbal Medicine and Graduate Institute of Healthy Industry Technology, College of Human Ecology, Chang Gung University of Science and Technology, Taoyuan 333, Taiwan; 4 Department of Anesthesiology, Chang Gung Memorial Hospital, Taoyuan 333, Taiwan; 5 Department of Marine Biotechnology and Resources, National Sun Yat-sen University, Kaohsiung 804, Taiwan; 6 School of Pharmacy, College of Pharmacy, China Medical University, Taichung 404, Taiwan; 7 Chinese Medicine Research and Development Center, China Medical University Hospital, Taichung 404, Taiwan; 8 Center for Molecular Medicine, China Medical University Hospital, Taichung 404, Taiwan; 9 Graduate Institute of Natural Products, Kaohsiung Medical University, Kaohsiung 807, Taiwan; 10 Graduate Institute of Marine Biology, National Dong Hwa University, Pingtung 944, Taiwan

**Keywords:** 9,11-secosterol, gorgonian, *Pinnigorgia*, anti-inflammatory, superoxide anion, elastase

## Abstract

Pinnigorgiols D (**1**) and E (**2**), two new 9,11-secosterols with a rearranged carbon skeleton, were isolated from a Taiwan gorgonian *Pinnigorgia* sp. The structures of these two compounds were elucidated on the basis of spectroscopic methods and were proven to possess a tricyclo[5,2,1,1]decane ring. The new secosterols **1** and **2** displayed significant inhibitory effects on the generation of superoxide anions and the release of elastase by human neutrophils.

## 1. Introduction

**Figure 1:**
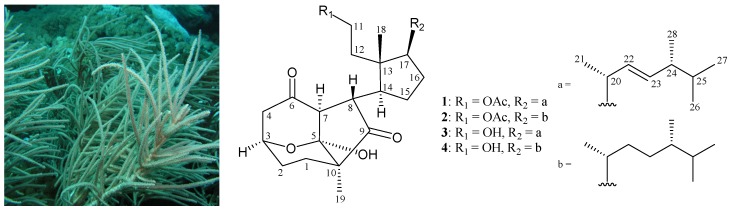
Gorgonian coral *Pinnigorgia* sp. and the structures of pinnigorgiols D (**1**), E (**2**), A (**3**), and B (**4**).

Gorgonian corals belonging to the genus *Pinnigorgia* (Family Gorgoniidae), have proven to be rich sources of bioactive sterols [1,2,3,4]. Previous bioassay results of these sterol analogues have demonstrated cytotoxic and anti-inflammatory activities. Following the above investigations, with the aim of discovering bioactive substances for new drug development in the future, we continue here to carry out an investigation on a Taiwan gorgonian *Pinnigorgia* sp., and two novel 9,11-secosterols, pinnigorgiols D (**1**) and E (**2**), with a rare carbon skeleton arrangement, were discovered (Figure 1). The ability of these two compounds to inhibit the generation of superoxide anions and the release of elastase in *N*-formyl methionyl leucylphenylalanine/cytochalasin B (fMLP/CB)-induced neutrophils were also evaluated.

## 2. Results and Discussion

Pinnigorgiol D (**1**) was obtained as an oil and had the molecular formula C_30_H_46_O_6_ as determined by high-resolution electrospray ionization mass spectrum (HRESIMS) at *m*/*z* 525.31883 (calcd. for C_30_H_46_O_6_ + Na, 525.31866), requiring eight degrees of unsaturation. The IR absorptions of **1** showed the presence of hydroxy (ν_max_ 3446 cm^−1^), ester (ν_max_ 1739 cm^−1^), and ketonic carbonyl (ν_max_ 1717 cm^−1^) groups. The ^13^C NMR and distortionless enhancement of polarization transfer (DEPT) data of **1** (Table 1) indicated the presence of 30 carbons, including seven methyls, seven sp^3^ methylenes, eight sp^3^ methines, a disubstituted double bond, and six quaternary carbons. The ^1^H NMR spectrum (Table 1) exhibited seven methyl signals at δ_H_ 2.06 (3H, s, acetate methyl), 1.12 (3H, s), 1.04 (3H, d, *J* = 7.2 Hz), 0.91 (3H, d, *J* = 6.8 Hz), 0.90 (3H, s), 0.83 (3H, d, *J* = 6.8 Hz), and 0.81 (3H, d, *J* = 6.8 Hz). The signal at δ_H_ 4.54 (1H, ddd, *J* = 11.6, 11.6, 4.4 Hz) and 3.86 (1H, ddd, *J* = 11.6, 11.6, 6.0 Hz) were assumed to be an oxymethylene group. It was found that the NMR signals of **1** were similar to those of a known 9,11-secosterol analogue, pinnigorgiol A (**3**) (Figure 1) [1], except that the signals corresponding to the 11-hydroxy group in **3** were replaced by signals for an acetoxy group in **1** (Table 2). The correlations from a nuclear Overhauser effect spectroscopy (NOESY) experiment of **1** also revealed that the stereochemistry of this metabolite was identical to that of **3**. Thus, pinnigorgiol D (**1**) was found to be the 11-*O*-acetyl derivative of **3**.

Pinnigorgiol E (**2**) was obtained as an oil and had the molecular formula C_30_H_48_O_6_ as determined by HRESIMS at *m/z* 527.33444 (calcd. for C_30_H_48_O_6_ + Na, 527.33431) and by analysis of NMR spectral data, requiring seven degrees of unsaturation. Initial analyses of the ^1^H and ^13^C NMR spectral data of **2** illustrated features very similar to those of **1** (Table 1) and a known secosterol, pinnigorgiol B (**4**) (Figure 1) [1], except that the signals corresponding to the 11-hydroxy group in **4** were replaced by signals for an acetoxy group in **2** (Table 3). Based on the above observations, pinnigorgiol E (**2**) was assigned as the 11-*O*-acetyl derivative of pinnigorgiol B (**4**).

**Figure 2:**
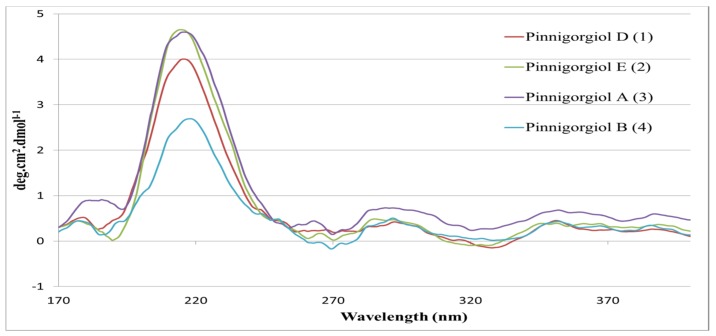
CD spectra of pinnigorgiols D (**1**), E (**2**), A (**3**), and B (**4**) [1].

The CD spectra of pinnigoriols D (**1**), E (**2**), A (**3**), and B (**4**) in methanol displayed positive Cotton effects at 216 nm (Δε = +4.0), 214 nm (Δε = +4.6), 216 nm (Δε = +4.6), and 218 nm (Δε = +2.7), respectively, [1] (Figure 2). These highlights confirmed that secosterols **1**–**4** possess the same configurations.

**Figure 3:**
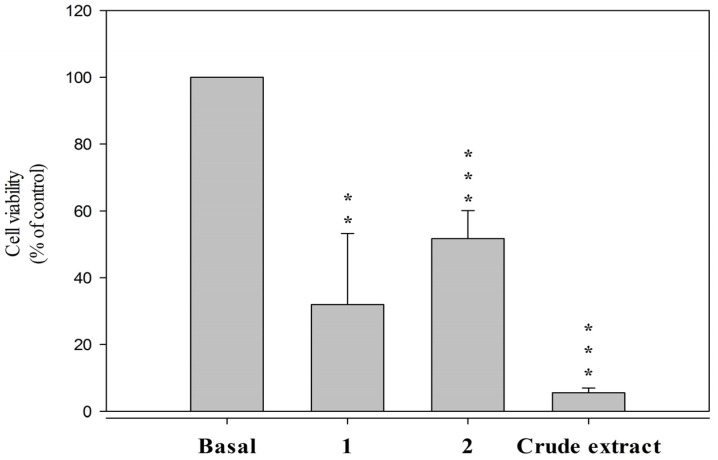
Secosterols **1** and **2** decreased viability of HSC-T6 in 10 μM for 24 h. Cells were treated with DMSO (control) and coral crude extract in 6 μg/mL. Cytotoxicity assay was monitored spectrophotometrically at 450 nm. Quantitative data are expressed as the mean ± S.E.M. (*n* = 3–4). ** *p* < 0.01, *** *p* < 0.001 compared to basal.

The hepatic stellate cell is the major cell type involved in liver fibrosis, which is the formation of scar tissue in response to liver damage. Secosterols **1** and **2** were tested against the HSC-T6 rat hepatic stellate cell line. Compounds **1** and **2** decreased the viability of HSC-T6 cells to 31.9% and 51.7%, respectively, at a concentration of 10 μM (Figure 3).

In a previous study, pinnigorgiols A (**3**) and B (**4**) were reported to significantly decrease the cell viability of HSC-T6 cells to 13.0% and 20.8%, respectively, at a concentration of 10 μM (Table 4) [1]. It seemed that the C-11 hydroxy group and the double bond between C-22/23 are critical for the cytotoxic activity of secosterols **1**–**4**.

The in vitro anti-inflammatory effects of secosterols **1** and **2** were tested. Pinnigorgiols D (**1**) and E (**2**) were found to exhibit inhibitory effects on the generation of superoxide anions (IC_50_ = 3.5 and 3.9 μM, respectively) and the release of elastase (IC_50_ = 2.1 and 1.6 μM, respectively) by human neutrophils (Table 5). Pinnigorgiols A (**3**) and B (**4**) were also found to display inhibitory effects on the generation of superoxide anions (IC_50_ = 4.0 and 2.5 μM, respectively) and the release of elastase (IC_50_ = 5.3 and 3.1 μM, respectively) [1]. Secosterol **2** show stronger activity in the inhibitory effect on the release of elastase, which indicated that an acetoxy substituent at C-11 and the absence of C-22/23 double bond would enhance the activity by comparison with the structure and anti-inflammatory data of **2** with those of **1**, **3**, and **4**.

## 3. Experimental Section

### 3.1. General Experimental Procedures

Optical rotations were measured on a Jasco P-1010 digital polarimeter (Japan Spectroscopic Corporation, Tokyo, Japan). CD spectra were recorded on a Jasco J-810 circular dichroism spectropolarimeter (Japan Spectroscopic Corporation, Tokyo, Japan). Infrared spectra were recorded on a Jasco FT/IR-4100 spectrometer (Japan Spectroscopic Corporation, Tokyo, Japan); peaks are reported in cm^−1^. The NMR spectra were recorded on a Varian Mercury Plus 400 spectrometer, using the residual CHCl_3_ signal (δ_H_ 7.26 ppm) as an internal standard for ^1^H NMR and CDCl_3_ (δ_C_ 77.1 ppm) for ^13^C NMR; coupling constants (J) are given in Hz. ESIMS and HRESIMS were recorded using a Bruker 7 Tesla solariX FTMS system (Bruker, Bremen, Germany). Column chromatography was performed on silica gel (230–400 mesh, Merck, Darmstadt, Germany). TLC was carried out on precoated Kieselgel 60 F_254_ (0.25 mm, Merck, Darmstade, Germany); spots were visualized by spraying with 10% H_2_SO_4_ solution followed by heating. Normal-phase HPLC (NP-HPLC) was performed using a system comprised of a Hitachi L-7110 pump (Hitachi Ltd., Tokyo, Japan) and a Rheodyne 7725 injection port (Rheodyne LLC, Rohnert Park, CA, USA). A semi-preparative normal-phase column (Supelco Ascentis Si Cat #:581515-U, 25 cm × 21.2 mm, 5 μm, Sigma-Aldrich, St. Louis, MO, USA) was used for NP-HPLC. Reversed-phase HPLC (RP-HPLC) was performed using a system comprised of a Hitachi L-2130 pump (Hitachi Ltd., Tokyo, Japan), a Hitachi L-2455 photodiode array detector (Hitachi Ltd., Tokyo, Japan), and a Rheodyne 7725 injection port (Rheodyne LLC., Rohnert Park, CA, USA). A reverse phase column (Luna^®^ 5 μm C18(2) 100 Å, AXIA Packed, 25 cm × 21.2 mm, Phenomenex Inc., Torrance, CA, USA) was used for RP-HPLC.

### 3.2. Animal Material

Specimens of the gorgonian corals *Pinnigorgia* sp. were collected by hand via scuba off the coast of Green Island, Taiwan, in August 2012 and stored in a freezer until extraction. A voucher specimen (NMMBA-TW-GC-2012-130) was deposited in the National Museum of Marine Biology & Aquarium, Taiwan. This organism was identified by a comparison with previous descriptions [5].

### 3.3. Extraction and Separation

Sliced bodies of *Pinnigorgia* sp. (wet weight 1.98 kg; dry weight 0.86 kg) were extracted with ethyl acetate (EtOAc) at room temperature. The EtOAc extract (84.9 g) was partitioned between methanol (MeOH) and *n*-hexane. The MeOH layer (12.6 g) was separated on Sephadex LH-20 and eluted using a mixture of dichloromethane (DCM) and MeOH (1:1) to yield 7 subfractions A–F. Fraction F was separated by silica gel column chromatography and eluted using *n*-hexane/acetone (stepwise, 1:1–pure acetone) to afford eight subfractions F1–F8. Fraction F2 was purified by silica gel column chromatography and eluted using *n*-hexane/acetone (stepwise, 9:1–pure acetone) to yield 13 subfractions F2A–F2M. Fraction F2D was purified by NP-HPLC using a mixture of *n*-hexane/EtOAc (3:1) to yield 17 subfractions F2D1–F2D17. Fraction F2D15 was purified by RP-HPLC, using a mixture of MeOH/H_2_O (95:5) to yield **1** (6.5 mg) and **2** (8.7 mg), respectively.

Pinnigorgiol D (**1**): colorless oil; ${\left[\alpha \right]}_{D}^{25}$ −6 (*c* 0.44, CHCl_3_); IR (neat) ν_max_ 3446, 1739, 1717 cm^−^^1^; ^1^H and ^13^C NMR data, see Table 1; ESIMS *m*/*z* 525 [M + Na]^+^; HRESIMS *m*/*z* 525.31883 (calcd. for C_30_H_46_O_6_ + Na, 525.31866).

Pinnigorgiol E (**2**): colorless oil; ${\left[\alpha \right]}_{D}^{25}$ −4 (*c* 0.33, CHCl_3_); IR (neat) ν_max_ 3446, 1734, 1717 cm^−^^1^; ^1^H and ^13^C NMR data, see Table 1; ESIMS *m*/*z* 527 [M + Na]^+^; HRESIMS *m*/*z* 527.33444 (calcd. for C_3__0_H_48_O_6_ + Na, 527.33431).

### 3.4. Anti-Hepatofibric Assay

The anti-hepatofibric effects of tested secosterols **1** and **2** were assayed using a WST-1 assay method. Anti-hepatofibric assays were carried out according to procedures described previously [6].

### 3.5. Generation of Superoxide Anions and Release of Elastase by Human Neutrophils 

Human neutrophils were obtained by means of dextran sedimentation and Ficoll centrifugation. Measurements of superoxide anion generation and elastase release were carried out according to previously described procedures [7,8]. Briefly, superoxide anion production was assayed by monitoring the superoxide dismutase-inhibitable reduction of ferricytochrome *c*. Elastase release experiments were performed using MeO–Suc–Ala–Ala–Pro–Valp–nitroanilide as the elastase substrate.

## 4. Conclusions

Pinnigorgiols D (**1**) and E (**2**) are rare sterols containing a tricyclo[5,2,1,1]decane ring in their structures. Prior to this study, only four compounds of this type had been isolated from sea hare *Aplysia kurodai* [9] and gorgonian coral *Pinnigorgia* sp. [1]. In an anti-inflammatory activity test, secosterol **2** displayed significantly inhibitory effects on the release of elastase by human neutrophils and may become lead compounds in future marine anti-inflammatory drug development [10,11]. The gorgonian coral *Pinnigorgia* sp. will be transplanted to culturing tanks located in the National Museum of Marine Biology & Aquarium, Taiwan, for the extraction of additional natural products to establish a stable supply of bioactive material.

## Figures and Tables

**Table 1:** ^1^H (400 MHz, CDCl_3_) and ^13^C (100 MHz, CDCl_3_) NMR data for secosterols **1** and **2**.

Position	1		2	
	δ_H_ (*J* in Hz)	δ_C_, Multiple	δ_H_ (*J* in Hz)	δ_C_, Multiple
1	1.38 m; 1.25 m	26.5, CH_2_	1.38 m; 1.25 m	26.6, CH_2_
2	2.02 m; 1.59 m	23.8, CH_2_	2.03 m; 1.59 m	24.1, CH_2_
3	4.65 br s	70.2, CH	4.65 dd (6.0, 5.2)	70.2, CH
4	2.91 dd (16.4, 6.8); 2.39 d (16.4)	44.1, CH_2_	2.91 dd (16.4, 6.8); 2.39 d (16.4)	44.0, CH_2_
5		101.4, C		101.4, C
6		207.7, C		207.7, C
7	3.04 s	59.6, CH	3.05 s	59.9, CH
8	3.18 dd (10.0, 2.0)	48.2, CH	3.19 dd (10.0, 2.0)	48.0, CH
9		216.6, C		216.6, C
10		49.4, C		49.5, C
11	4.54 ddd (11.6, 11.6, 4.4)	61.2, CH_2_	4.52 ddd (11.6, 11.6, 4.8)	61.3, CH_2_
	3.86 ddd (11.6, 11.6, 6.0)		3.87 ddd (11.6, 11.6, 6.0)	
12	1.89 m; 1.67 m	36.1, CH_2_	1.89 m; 1.61 m	36.1, CH_2_
13		46.7, C		46.8, C
14	2.04 m	45.8, CH	2.04 m	46.1, CH
15	1.94 m; 1.77 m	27.3, CH_2_	1.92 m; 1.80 m	27.0, CH_2_
16	1.98 m; 1.45 m	24.0, CH_2_	2.02 m; 1.42 m	24.2, CH_2_
17	1.43 m	50.0, CH	1.42 m	49.8, CH
18	0.90 s	17.0, CH_3_	0.90 s	16.8, CH_3_
19	1.12 s	12.1, CH_3_	1.12 s	12.1, CH_3_
20	2.27 m	36.8, CH	1.45 m	33.3, CH
21	1.04 d (7.2)	22.6, CH_3_	1.04 d (7.2)	20.4, CH_3_
22	5.26 dd (15.6, 7.6)	133.7, CH	1.45 m; 0.90 m	32.6, CH_2_
23	5.23 dd (15.6, 7.6)	133.3, CH	1.37 m; 0.90 m	31.8, CH_2_
24	1.89 m	43.1, CH	1.21 m	39.0, CH
25	1.48 m	33.1, CH	1.56 m	31.5, CH
26	0.83 d (6.8)	20.0, CH_3_	0.78 d (6.8)	17.6, CH_3_
27	0.81 d (6.8)	19.7, CH_3_	0.85 d (6.8)	20.4, CH_3_
28	0.91 d (6.8)	17.5, CH_3_	0.77 d (6.8)	15.5, CH_3_
11-OAc		172.5, C		172.4, C
	2.06 s	21.2, CH_3_	2.05 s	21.2, CH_3_

**Table 2:** NMR data for the 11-acetoxy component in pinnigorgiol D (**1**), and the 11-hydroxy component in pinnigorgiol A (**3**).

Position	1		3 ^a^	
	δ_H_ (*J* in Hz)	δ_C_, Multiple	δ_H_ (*J* in Hz)	δ_C_, Multiple
11	4.54 ddd (11.6, 11.6, 4.4)	61.2, CH_2_	3.83 m	59.0, CH_2_
	3.86 ddd (11.6, 11.6, 6.0)			
12	1.89 m; 1.67 m	36.1, CH_2_	1.89 dt (15.5, 5.5); 1.74 m	39.5, CH_2_
13		46.7, C		46.6, C
11-OAc		172.5, C		
	2.06 s	21.2, CH_3_		

^a^ Data was reported by Chang et al. [1].

**Table 3:** NMR data for the 11-acetoxy component in pinnigorgiol E (**2**), and the 11-hydroxy component in pinnigorgiol B (**4**).

Position	2		4 ^a^	
	δ_H_ (*J* in Hz)	δ_C_, Multiple	δ_H_ (*J* in Hz)	δ_C_, Multiple
11	4.52 ddd (11.6, 11.6, 4.8)	61.3, CH_2_	3.81 m	59.1, CH_2_
	3.87 ddd (11.6, 11.6, 6.0)			
12	1.89 m; 1.61 m	36.1, CH_2_	1.89 ddd (16.0, 6.0, 5.2); 1.69 m	39.7, CH_2_
13		46.8, C		46.6, C
11-OAc		172.4, C		
	2.05 s	21.2, CH_3_		

^a^ Data was reported by Chang et al. [1].

**Table 4:** Secosterols **1**–**4** decreased viability of HSC-T6 in 10 μM for 24 h.

Compound	1	2	3 ^a^	4 ^a^
Inhibition rate (% of basal)	31.9	51.7	13.0	20.8

^a^ Data was reported by Chang et al. [1].

**Table 5:** Inhibitory effects of secosterols **1**–**4** on the generation of superoxide anions and the release of elastase by human neutrophils in response to fMet-Leu-Phe/cytochalastin B (fMLP/CB).

Compound	Superoxide Anion	Elastase Release
	IC_50_ (μM)	IC_50_ (μM)
**1**	3.5	2.1
**2**	3.9	1.6
**3** ^a^	4.0	5.3
**4** ^a^	2.5	3.1

^a^ Data was reported by Chang et al. [1].
